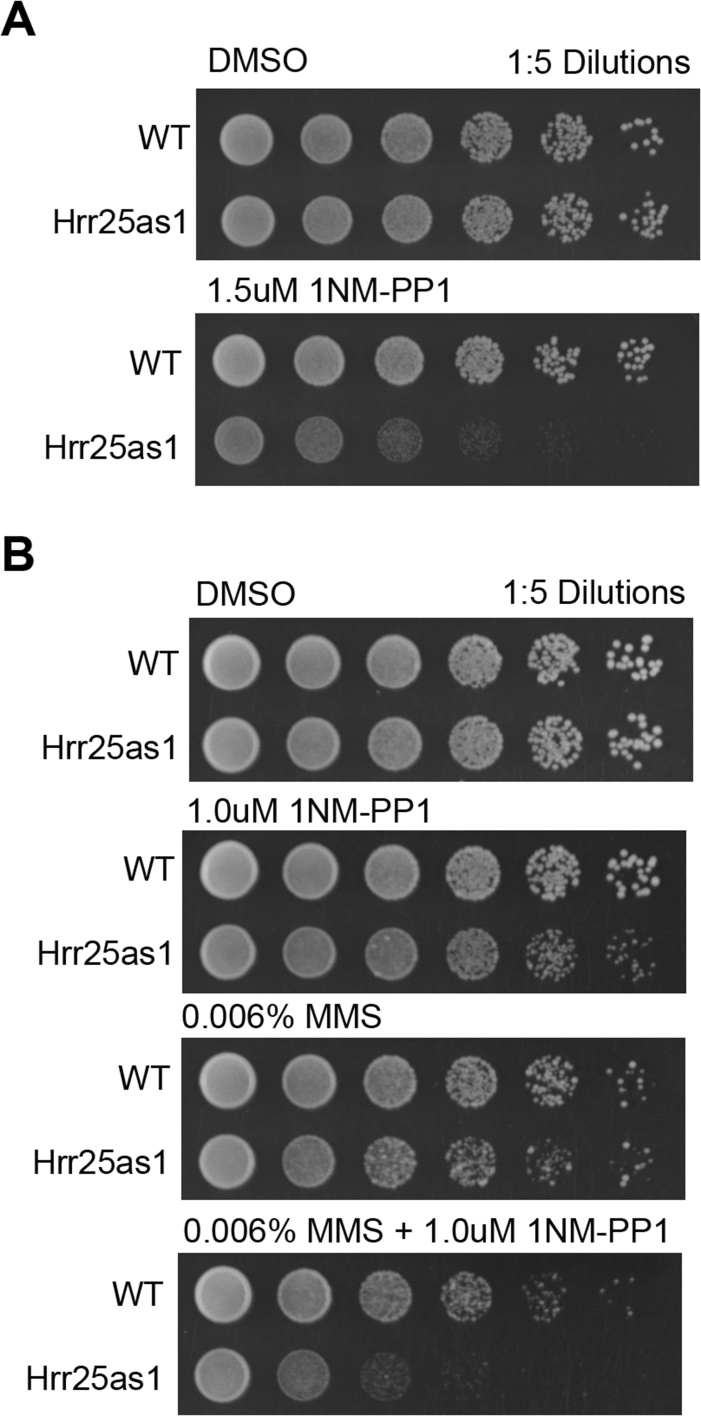# Profiling Tel1 signaling reveals a non-canonical motif targeting DNA repair and telomere control machineries

**DOI:** 10.1016/j.jbc.2025.108194

**Published:** 2025-01-16

**Authors:** William J. Comstock, Shrijan Bhattarai, Ethan J. Sanford, Marcos V.A.S. Navarro, Marcus B. Smolka

**Affiliations:** 1 Department of Molecular Biology and Genetics, Weill Institute for Cell and Molecular Biology, Cornell University, Ithaca, New York, USA; 2 IFSC Institute of Physics of São Carlos, University of São Paulo, São Carlos, São Paulo, Brazil

**Keywords:** DNA repair, phosphoproteomics, mass spectrometry, kinase signaling, telomere maintenance, phosphorylation, Tel1, DNA damage signaling

## Abstract

The stability of the genome relies on phosphatidyl inositol 3-kinase-related kinases (PIKKs) that sense DNA damage and trigger elaborate downstream signaling responses. In *Saccharomyces cerevisiae*, the Tel1 kinase (ortholog of human ATM) is activated at DNA double-strand breaks (DSBs) and short telomeres. Despite the well-established roles of Tel1 in the control of telomere maintenance, suppression of chromosomal rearrangements, activation of cell cycle checkpoints, and repair of DSBs, the substrates through which Tel1 controls these processes remain incompletely understood. Here we performed an in-depth phosphoproteomic screen for Tel1-dependent phosphorylation events. To achieve maximal coverage of the phosphoproteome, we developed a scaled-up approach that accommodates large amounts of protein extracts and chromatographic fractions. Compared to previous reports, we expanded the number of detected Tel1-dependent phosphorylation events by over 10-fold. Surprisingly, in addition to the identification of phosphorylation sites featuring the canonical motif for Tel1 phosphorylation (S/T-Q), the results revealed a novel motif (D/E-S/T) highly prevalent and enriched in the set of Tel1-dependent events. This motif is unique to Tel1 signaling and not shared with the Mec1 kinase, providing clues to how Tel1 plays specialized roles in DNA repair and telomere length control. Overall, these findings define a Tel1-signaling network targeting numerous proteins involved in DNA repair, chromatin regulation, and telomere maintenance that represents a framework for dissecting the molecular mechanisms of Tel1 action.

Phosphatidyl Inositol 3-Kinase-related Kinases (PIKKs) play key roles in genome maintenance by sensing DNA damage and orchestrating DNA damage response signaling networks. In *Saccharomyces cerevisiae*, the PIKKs Mec1 (human ATR) and Tel1 (human ATM) play central roles in coordinating DNA damage responses. While Mec1 senses and responds to single-stranded DNA (ssDNA) *via* Ddc2 (human ATRIP), Tel1 associates with the blunt ends of double-stranded DNA breaks (DSBs) and short telomeres (1, 2, 3) (Fig. 1*A*). Recruitment of either Mec1 or Tel1 to DNA lesions promotes the activation of those kinases, which then initiate a signaling cascade that includes phosphorylation of downstream DNA damage checkpoint kinases Dun1, Rad53, and Chk1 (4, 5, 6). Mec1 and Tel1 play partially redundant roles in the orchestration of DNA damage responses. For example, both kinases are important for the suppression of gross chromosomal rearrangements (GCRs), with GCRs accumulating synergistically upon deletion of both kinases (7). Mec1 also plays key roles in surveilling and controlling DNA replication and replication stress responses, most of which are not shared with Tel1 (8). On the other hand, Tel1 plays a crucial role in telomere maintenance by binding to short telomeres during late S/G2 and promoting their elongation (1, 3). Moreover, Tel1 is also involved in meiotic DSB interference (9). The downstream targets through which Tel1 controls these processes and its other related roles in DSB response and repair remain incompletely understood. Tel1 is activated by recruitment to blunt DNA ends *via* the MRX complex, composed of Mre11, Rad50, and Xrs2 (9, 10). This complex is rapidly recruited to DSBs and plays roles in DNA end-recognition and end-processing. Within the MRX complex, Xrs2 is responsible for interacting with and activating Tel1 *via* its C-terminal Tel1-interacting region (11).

**Figure 1:**
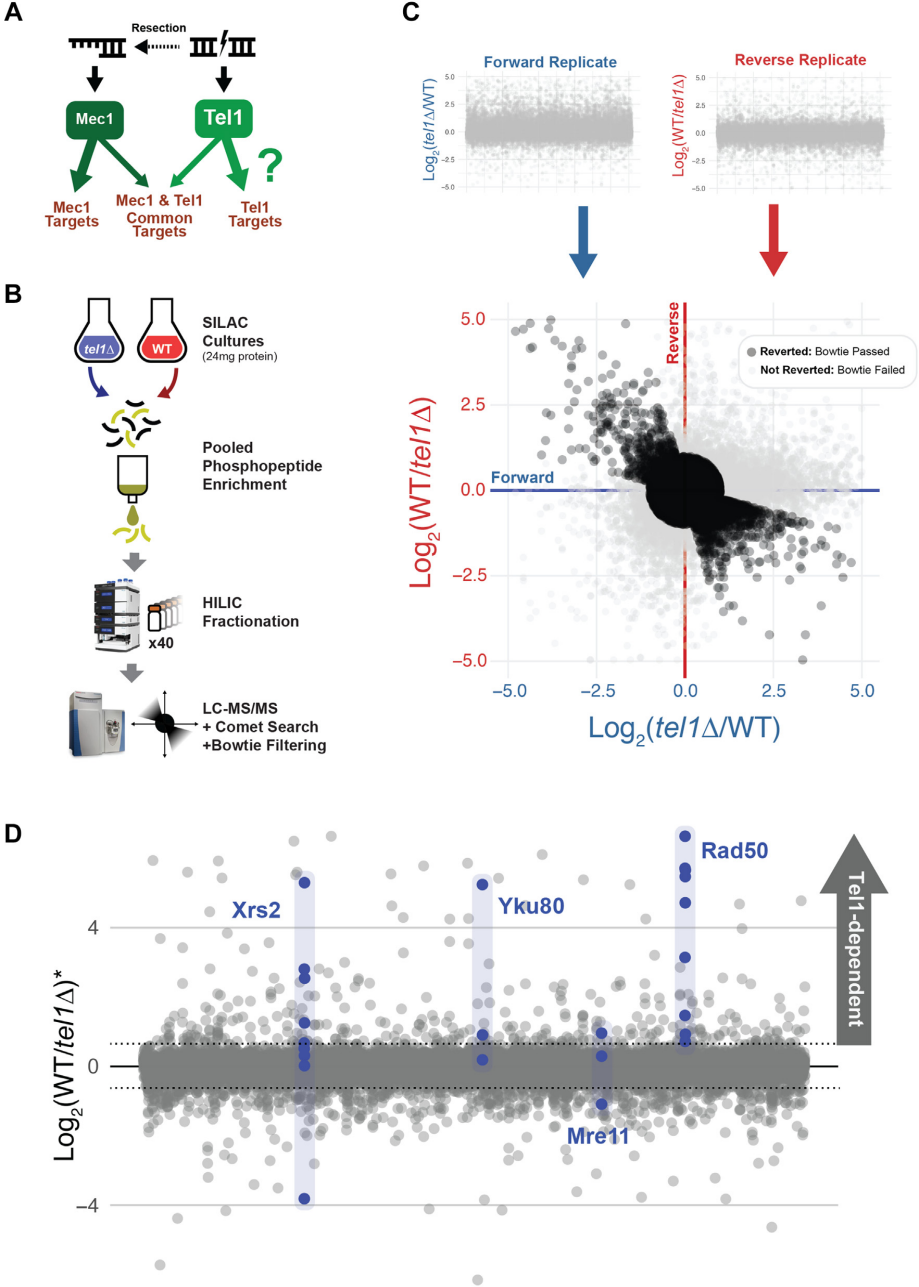
**Large-scale phosphoproteomics of Tel1-dependent phosphorylation**. *A*, overview of DNA lesions activating PIKKs, with Mec1 and Tel1 canonically becoming activated by different forms of DNA damage. *B*, a schematic of the large-scale phosphoproteomic pipeline. Large SILAC yeast cultures are required to obtain sufficient starting material for tryptic digestion and phosphopeptide enrichment. Extensive HILIC prefractionation reduces sample complexity and allows for in-depth LC-MS/MS analysis, followed by database searching and Bowtie replicate filtering. *C*, Bowtie filtering of forward and reverse replicates performed for each experimental condition, in which experimental conditions are inverted between SILAC channels. The replicates are then plotted against one another to determine which phosphopeptides feature appropriately inverting quantitative ratios, discarding any that do not invert. *D*, plot depicting averaged ratios for 17,401 phosphopeptides that passed Bowtie filtering. Among these are phosphopeptides on Yku80 and components of the MRX complex, most of which are found to be Tel1-dependent. *Asterisk* indicates an average of the quantitative ratios of both the forward and reverse replicates.

Mec1 and Tel1 are well known to preferentially phosphorylate motifs featuring serine or threonine residues followed by a glutamine residue (S/T-Q motif) (2). Several phosphoproteomic screens have been performed to map the signaling network mediated by Mec1, which led to the identification of hundreds of putative Mec1 targets phosphorylated at the S/T-Q motif (12, 13, 14). Comparatively, a much smaller set of Tel1 targets was identified, consistent with the notion that Tel1 contributes less to the DNA damage signaling response relative to Mec1. Our previous phosphoproteomic screen for Tel1 targets identified only 4 highly Tel1-dependent phosphorylation events, including Rad50, Enp1, Asg1, and Tfg1 (14). Other known Tel1 targets include Cdc13, Rad53, and Pol4 (15, 16).

In an effort to map the Tel1 signaling network with higher coverage, we deployed a scaled-up phosphoproteomic pipeline alongside our previously published “bowtie” filtering methodology (17). *tel1Δ* yeast strains were compared against wild-type yeast using stable isotope labeling by amino acids in cell culture (SILAC) mass spectrometry, from which hundreds of novel Tel1-dependent sites were detected. As expected, a significant proportion of these sites featured the canonical S/T-Q motif. Surprisingly, we also detected a strong prevalence and enrichment of a novel acidophilic consensus motif (D/E-S/T) among Tel1-dependent phosphorylation events. Overall, our results define a Tel1-signaling network targeting proteins involved in DNA repair, chromatin, and telomere maintenance that provides a framework for dissecting the molecular mechanisms of Tel1 action.

## Results

### Large-scale phosphoproteomic analysis of Tel1-dependent signaling

**Figure S1:**
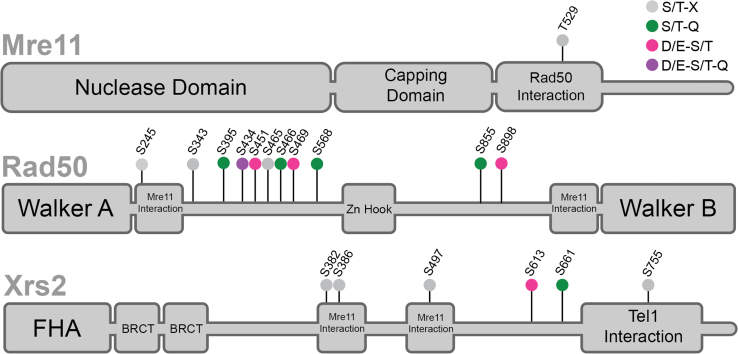


Our previous phosphoproteomic screen of the Mec1 and Tel1 signaling networks in budding yeast resulted in the identification of a small set of Tel1-dependent phosphorylation events (14). In order to map the Tel1-signaling network with greater depth, we employed a scaled-up phosphoproteomic pipeline (Fig. 1*B*). This pipeline involved scaling up yeast cultures to obtain at least 12 mg of extracted protein per experimental condition, followed by multiple IMAC phosphopeptide enrichments in parallel and extensive sample prefractionation (40 fractions) *via* hydrophilic interaction liquid chromatography (HILIC) prior to LC-MS/MS analysis. Yeast cultures were grown in SILAC media for incorporation of light or heavy amino acids that were used for relative quantification. To increase confidence in the identification, quantification, and phosphorylation site assignment, we used a Bowtie filtering method in which the SILAC channels were reversed (“forward” and “reverse” replicates) in the second replicate of every phosphoproteomic analysis (17). Cultures of wild-type and *tel1Δ* cells were treated with 0.1% methyl methanesulfonate (MMS) for 90 min to induce DSBs. Experiments were performed swapping heavy and light amino acids for each genotype, after which these forward and reverse replicates were plotted against one another and their ratios averaged (Fig. 1*C*). In total, 22,960 phosphopeptides were detected, of which 17,401 passed the Bowtie filtering and 442 phosphorylation sites and clusters were found to be Tel1-dependent (with at least 50% reduction in abundance in *tel1Δ* cells compared to wild type) (Fig. 1*D*). As expected, components of the MRX complex and DSB repair protein YKU80 were found to contain several Tel1-dependent phosphorylation sites, validating our dataset (Figs. 1*D* and S1).

### Tel1-dependent phosphorylation is enriched in S/T-Q motifs on proteins involved in chromatin and chromosome biology

**Figure 2:**
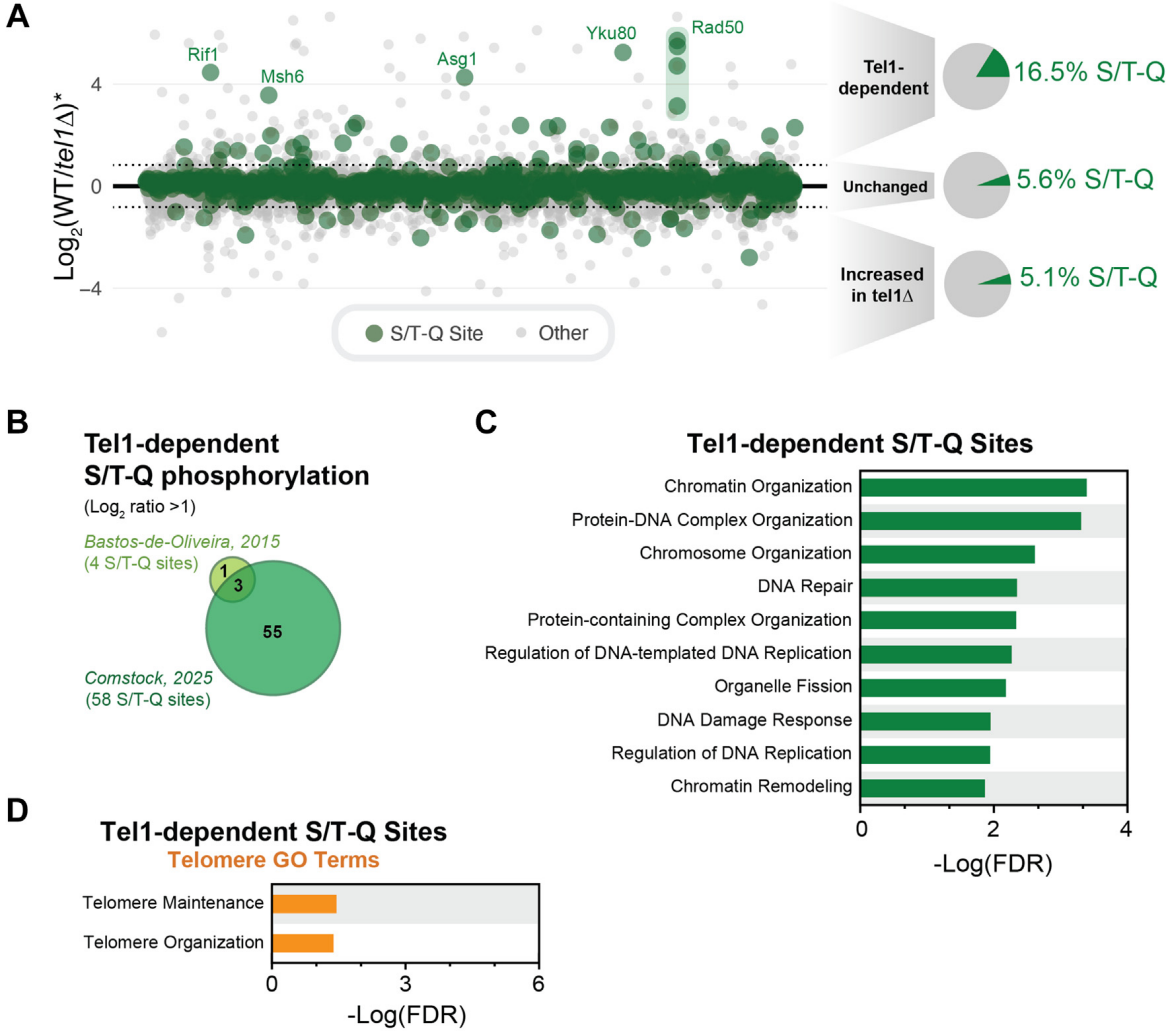
**The S/T-Q motif is enriched among Tel1-dependent phosphorylation events on proteins involved in the DNA damage response and telomere maintenance.***A*, plot depicting the averaged ratio for 17,401 phosphopeptides that passed Bowtie filtering. S/T-Q sites are highlighted in *green*. Tel1-dependent phosphorylation events are over 3X enriched for the canonical preferred PIKK S/T-Q motif when compared to unchanged phosphorylation events. *Asterisk* indicates an average of the quantitative ratios of both the forward and reverse replicates. *B*, comparison of the number of Tel1-dependent phosphorylation events detected in the current study *versus* our previous investigation of the Tel1 signaling network. *C*, the top 10 GO terms (minus terms containing “process”) enriched among proteins featuring Tel1-dependent S/T-Q sites. *D*, telomere-related GO term enrichment among proteins featuring Tel1-dependent S/T-Q sites.

**Figure S2:**
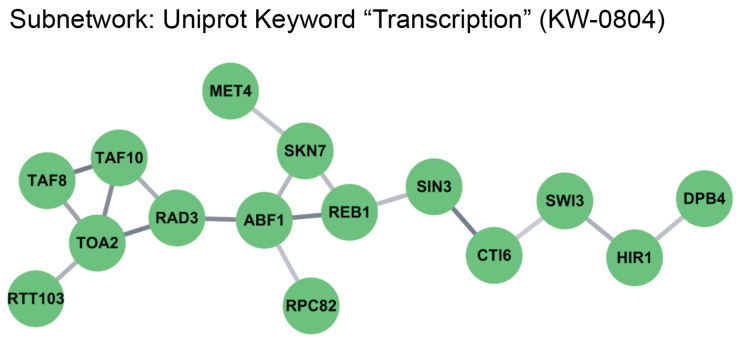


Consistent with the known preferred motif for Tel1 phosphorylation, there was an enrichment for the S/T-Q motif among the set of Tel1-dependent sites uncovered. 16.5% of Tel1-dependent sites were found to feature this motif compared to 5.6% of the group of phosphorylation sites not affected by *TEL1* deletion (Fig. 2*A*). With the identification of 58 Tel1-dependent phosphorylation sites featuring this S/T-Q motif (63 including clusters), the number of Tel1-dependent S/T-Q sites has been expanded 14-fold over our previous phosphoproteomic screen for Tel1 targets (Fig. 2*B*). GO term analysis revealed an enrichment in proteins involved in chromatin and chromosome organization (Fig. 2*C*) (18). Proteins featuring Tel1-dependent phosphorylation at S/T-Q sites also included proteins with established roles in DNA repair and telomere maintenance, such as Msh6, Rif1, Yku80, and Rad50 (Fig. 2, *A* and *D*), and represent potential mechanistic links between Tel1 and its roles in controlling telomere length. Tel1-dependent phosphorylation events at the S/T-Q motif were also present on proteins implicated in transcription, indicating potential roles for canonical Tel1 signaling in transcriptional regulation (Fig. S2) (19).

### A novel D/E-S/T motif is enriched among Tel1-dependent phosphorylation events

**Figure 3:**
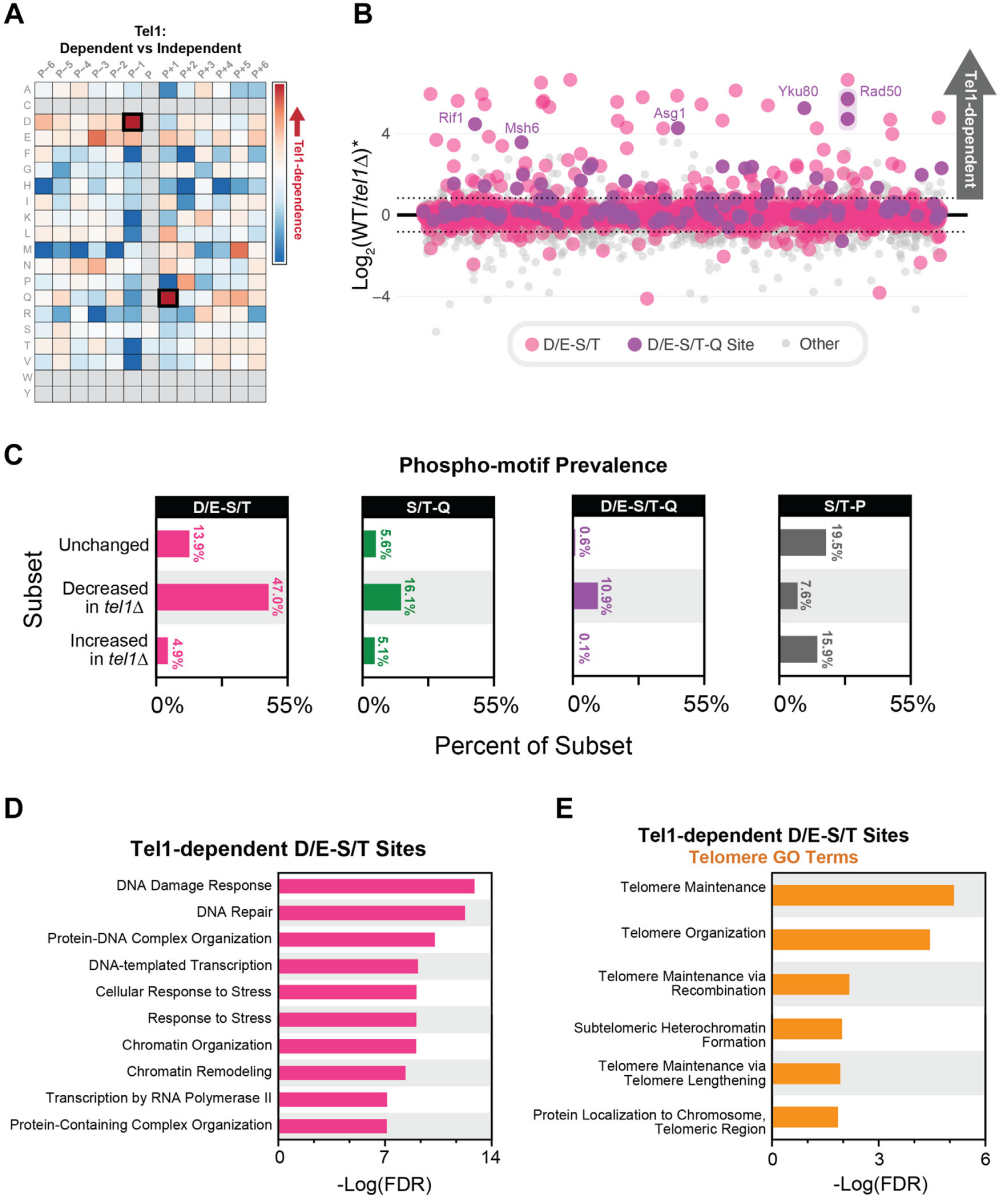
**A novel D/E-S/T motif is enriched among Tel1-dependent phosphorylation events.***A*, heatmap illustrating enrichment of amino acid residues at loci relative to phosphorylation events found to be Tel1-dependent. Acidic residues, particularly aspartic acid, are found to be enriched in the −1 locus. Enrichment for the S/T-Q motif is also illustrated here. *B*, plot depicting the averaged ratio for 17,401 phosphopeptides that passed Bowtie filtering with D/E-S/T sites highlighted in *pink* and the sites with the combined motif (D/E-S/T-Q) highlighted in *purple*. *Asterisk* indicates an average of the quantitative ratios of both the forward and reverse replicates. *C*, prevalence of four phospho-motifs among subsets of the Tel1 signaling dataset. The most enriched motif in phosphopeptides that decreased upon deletion of Tel1 was D/E-S/T, with S/T-Q and D/E-S/T-Q motifs also showing high enrichment among this same subset of the data. *D*, the top 10 GO terms (minus terms containing “process”) enriched among proteins featuring Tel1-dependent D/E-S/T sites. *E*, telomere-related GO term enrichment among proteins featuring Tel1-dependent D/E-S/T sites.

**Figure S3:**
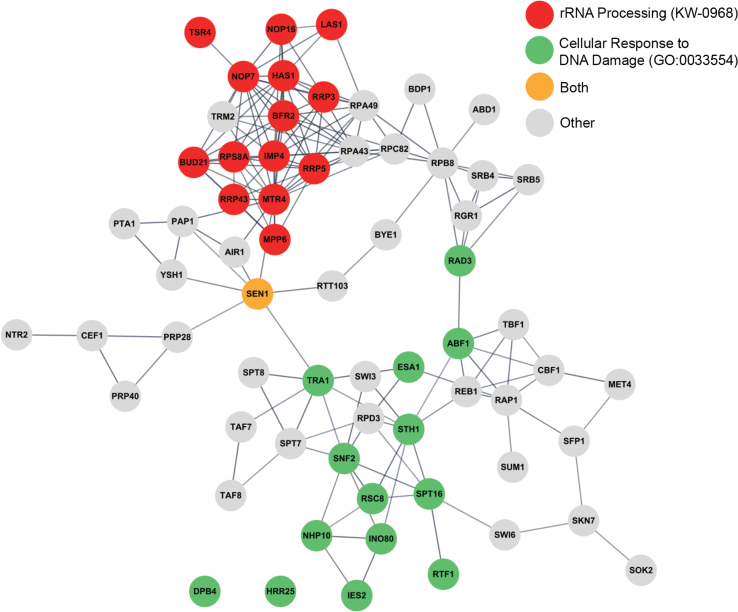


Unexpectedly, amino acid residue enrichment analysis surrounding the Tel1-dependent sites revealed a strong enrichment for an acidic residue at the −1 position relative to the phosphorylation site (Fig. 3*A*). Enrichment analysis revealed that the D/E-S/T motif represented nearly 50% of the Tel1-dependent sites identified, a higher proportion compared to the ∼16% comprised of the S/T-Q motif (Fig. 3, *B* and *C*). Notably, S/T-Q sites preceded by an acidic residue (D/E-S/T-Q motif) were drastically enriched in the set of Tel1-dependent sites, displaying an almost 20-fold increase in prevalence compared to the set of sites not altered by *TEL1* deletion, suggesting a strong preference for this motif by Tel1 (Fig. 3*C*). The set of Tel1-dependent phosphorylation events at the D/E-S/T motif was strongly enriched for proteins involved in the DNA damage response and DNA repair (Fig. 3*D*) and contained more GO terms related to telomere biology compared to the set of S/T-Q motifs (Figs. 3*E* and 2*D*). These results show that the proteins with phosphorylation at the D/E-S/T motif tend to align with known Tel1 functions and suggest that this novel Tel1 signaling motif reflects phosphorylation events through which Tel1 exerts at least part of its regulatory roles. Proteins implicated in RNA metabolic processes were also prevalent among the 189 Tel1-dependent D/E-S/T phosphorylation sites and clusters, including a prominent connected node of proteins implicated in ribosomal RNA processing (Fig. S3).

### Mec1-dependent phosphorylation events do not feature D/E-S/T motif enrichment

**Figure 4:**
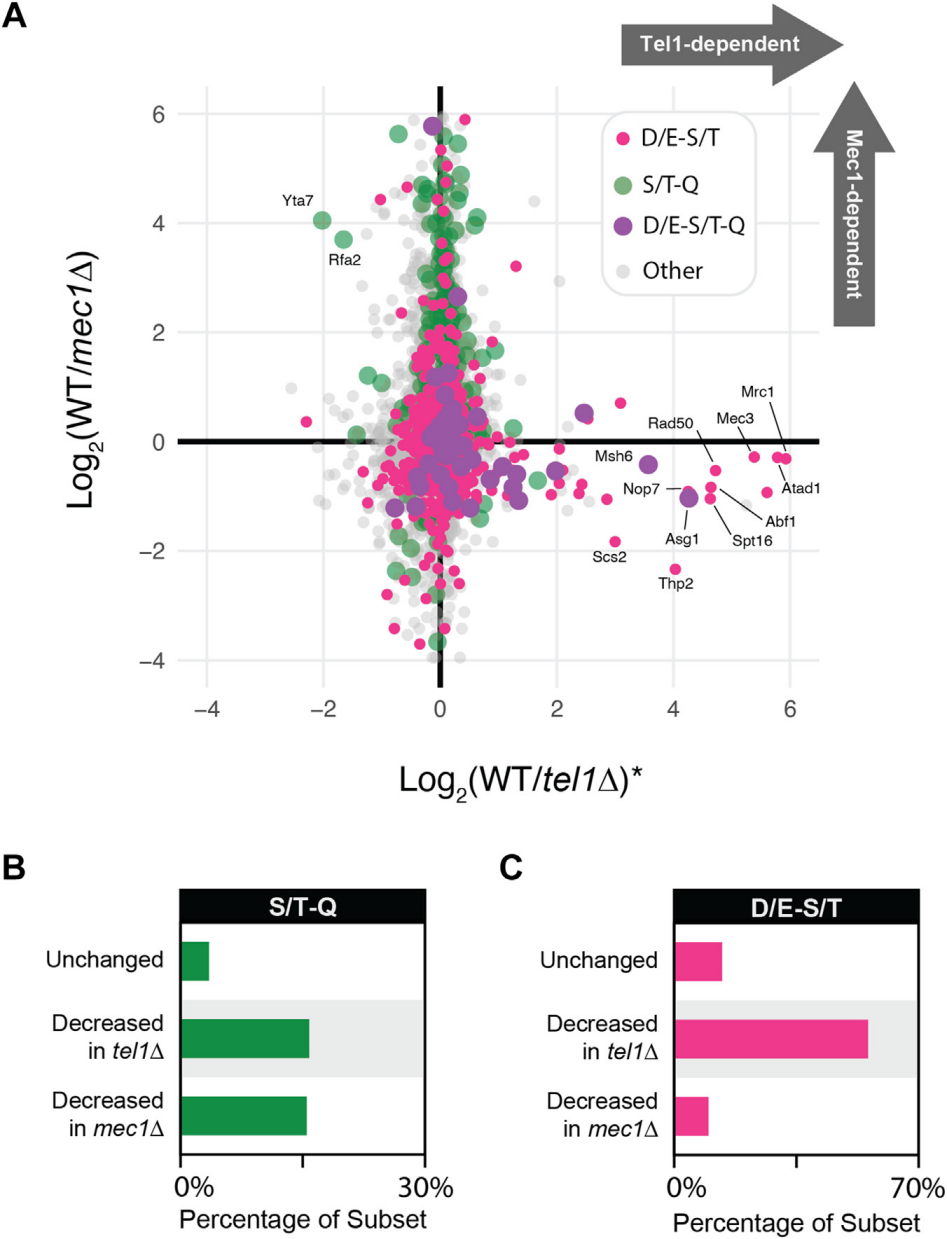
**The D/E-S/T motif is more enriched in Tel1-dependent phosphorylation events compared to Mec1-dependent phosphorylation events.***A*, scatter plot with phosphoproteomic data of Tel1 dependency plotted against phosphoproteomic data of Mec1 dependency. Increased abundance of Tel1-dependent phosphorylation events upon loss of Mec1 supports the notion that Tel1 engages in some amount of compensatory signaling when Mec1 activity is compromised. *Asterisk* indicates an average of the quantitative ratios of both the forward and reverse replicates. *B*, enrichment of the S/T-Q motif among Tel1- and Mec1-dependent phosphorylation events illustrates a higher enrichment for this motif among Mec1-dependent phosphorylation events. *C*, enrichment of the D/E-S/T motif among Tel1- and Mec1-dependent phosphorylation events strongly suggests that D/E-S/T motif enrichment is exclusive to Tel1-dependent signaling.

We next asked if enrichment of the D/E-S/T consensus motif is also observed in Mec1-dependent phosphorylation events or if it is a feature specific to Tel1-dependent signaling. Previous screens for Mec1-dependent phosphorylation events showed no such enrichment but also featured different treatment conditions and genetic backgrounds (20). Therefore, we performed a phosphoproteomic screen for Mec1-dependent phosphorylation events by comparing wild-type cells to *MEC1*-deleted cells with the same genetic background as our *TEL1*-deleted cells, both treated with 0.1% MMS (Fig. 4*A*). Interestingly, no enrichment for the D/E-S/T consensus motif was found among Mec1-dependent phosphorylation events, whereas the canonical S/T-Q phosphorylation motif was more strongly enriched among Mec1-dependent phosphorylation sites than it was among Tel1-dependent sites (Fig. 4, *B* and *C*). These findings reveal that the phosphorylation of D/E-S/T sites is a feature specific to Tel1 signaling. Notably, we observed that several Tel1 signaling events mapped here are induced in cells lacking Mec1 (lower-right quadrant in Fig. 4*A*), consistent with genetic data showing that in the absence of Mec1, Tel1 plays an important compensatory role in preventing GCRs, slow growth, and DNA damage sensitivity (8, 21).

### Tel1-dependent phosphorylation events at the D/E-S/T motif are not dependent on downstream kinases Dun1, Rad53, or Hrr25

**Figure 5:**
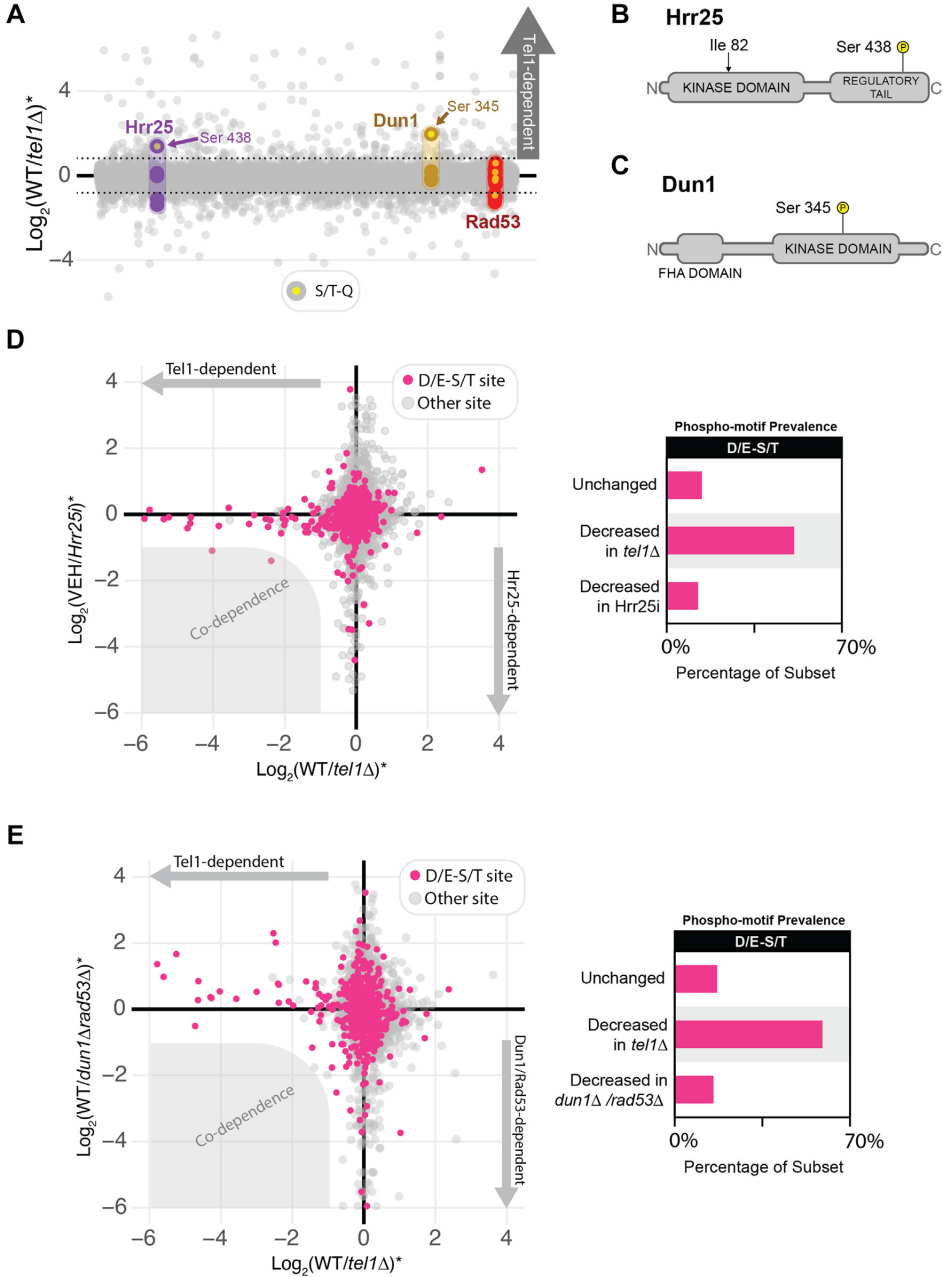
**Tel1-dependent D/E-S/T phosphorylation events are not dependent on downstream kinases Hrr25, Dun1, or Rad53.***A*, plot of phosphoproteomic data showing that S/T-Q sites on kinases Hrr25 and Dun1 were found to be Tel1-dependent. *Asterisk* indicates an average of the quantitative ratios of both the forward and reverse replicates. *B*, Sequence map of Hrr25 showing where an analog-sensitivity mutation was made (Ile 82) as well as the location of the Tel1-dependent S/T-Q phosphorylation event (Ser 438). *C*, sequence map of Dun1 showing the location of the Tel1-dependent S/T-Q phosphorylation event (Ser 345). *D*, scatter plot for phosphoproteomic data of Tel1 dependency plotted against phosphoproteomic data of Hrr25-dependency. No significant co-dependence of signaling events was observed. The D/E-S/T motif was not found to be enriched among Hrr25-dependent phosphorylation events. Hrr25 kinase inhibition was achieved by treating cultures with 1 micromolar 1NM-PP1. *E*, scatter plot for phosphoproteomic data of Tel1 dependency plotted against phosphoproteomic data of Dun1/Rad53 dependency. No significant co-dependence of signaling events was observed. The D/E-S/T motif was not found to be enriched among Dun1/Rad53-dependent phosphorylation events.

The Tel1-dependent phosphorylation events featuring the D/E-S/T motif may represent direct substrates of Tel1 or could be targeted by other downstream kinases under the control of Tel1. To determine the effect of downstream kinases on the phosphorylation status of Tel1-dependent phosphorylation events at the D/E-S/T motif, we searched for kinases containing Tel1-dependent phosphorylation in our dataset, which would point to kinases potentially activated by Tel1. This analysis revealed that the Hrr25 and Dun1 kinases are potential downstream kinases under the control of Tel1-dependent signaling (Fig. 5*A*). Consistent with this notion, Hrr25 and Dun1 contain a Tel1-dependent S/T-Q phosphorylation site at their regulatory and kinase domains, respectively (Fig. 5, *B* and *C*). We performed phosphoproteomic analyses at normal-scale in cells defective for these kinases to monitor the effect on Tel1-dependent phosphorylation events at the D/E-S/T motif. Given that Hrr25 is essential for cell survival, a documented ATP analog-sensitive mutation was created (Hrr25as) to allow for screening of Hrr25-dependent phosphorylation events (22). This system enables acute inhibition of Hrr25, which was confirmed by the observed increased cell sensitivity to both the ATP analog (1NM-PP1) and MMS in the presence of the analog (Fig. S4). Phosphoproteomic analysis revealed no enrichment for the D/E-S/T consensus motif in the set of Hrr25-dependent phosphorylation events (Fig. 5*D*). Importantly, none of the Tel1-dependent phosphorylation events at the D/E-S/T motif were found to be strongly reduced upon impairment of Hrr25 (no co-dependence). These results rule out Hrr25 as the kinase responsible for phosphorylating the Tel1-dependent phosphorylation events at the D/E-S/T motif.

We next tested if Dun1 was required for the phosphorylation of the Tel1-dependent phosphorylation events at the D/E-S/T motif. Notably, we did not detect Tel1-dependent phosphorylation on Rad53 (Fig. 5*A*), a kinase involved in the canonical activation of Dun1, consistent with previous reports suggesting that Dun1 might also be directly activated by Tel1 (6). Nonetheless, to rule out any contribution from Rad53 and/or Dun1, we utilized cells lacking both kinases in a normal-scale phosphoproteomic analysis. As with Hrr25, no enrichment for the D/E-S/T consensus motif was observed among Dun1/Rad53-dependent phosphorylation events (Fig. 5*E*). Interestingly, several Tel1-dependent events at the D/E-S/T motif were observed to be up-regulated upon double deletion of *DUN1* and *RAD53* (Fig. 5*E*), suggesting that in the absence of these kinases, Tel1 plays an important compensatory role in the DNA damage response, similar to what was observed in the absence of Mec1. Overall, these findings rule out the involvement of Dun1, Rad53, and Hrr25 as kinases downstream of Tel1 signaling responsible for the phosphorylation of Tel1-dependent phosphorylation sites at the D/E-S/T motif, strengthening the possibility that these sites are directly phosphorylated by Tel1.

### Mutating the Tel1 substrate recognition pocket selectively reduces Tel1-dependent phosphorylation at D/E-S/T motifs

**Figure 6:**
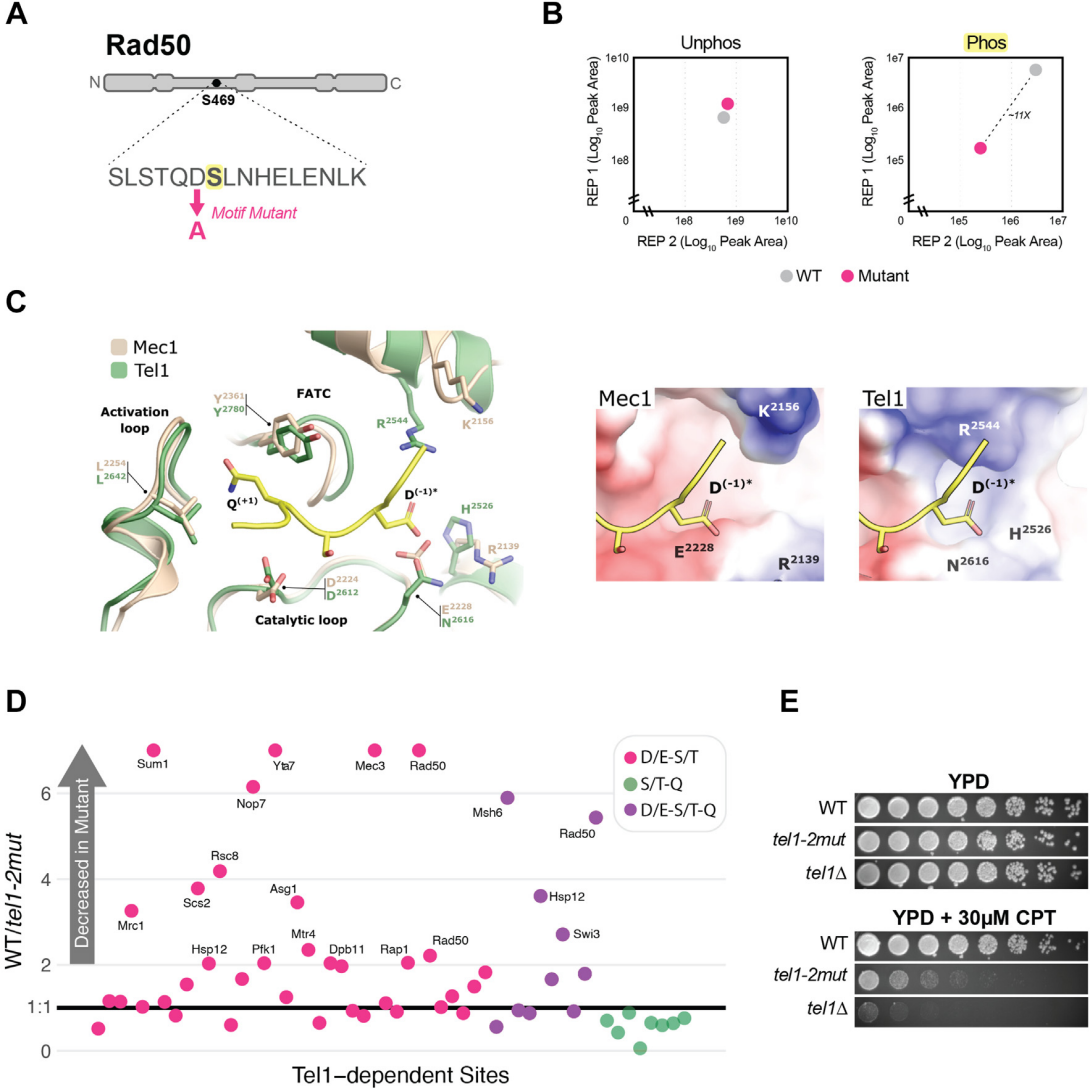
**Substrate and kinase mutations validate Tel1 preference for D/E-S/T motifs**. *A*, diagram illustrating the Tel1-dependent phosphorylation site at serine 469 on Rad50. The aspartic acid previous to this serine was mutated to alanine to assess the importance of a negative residue at the −1 position for Tel1-dependent phosphorylation. *B*, FLAG-tagged Rad50 with and without the motif mutation was immunoprecipitated and analyzed *via* mass spectrometry. Peak area quantification reveals a drop in phosphorylation of Rad50 serine 469 after motif mutation. *C*, structural superimposition of yeast Mec1 and Tel1 (PDB IDs 6S8F and 6Z2X respectively) highlighting the substrate-binding pocket and key residues involved in the recognition of peptide substrates *via* their positions −1 (Tel1 H^2526^, R^2544^, and N^2616^; Mec1 R^2139^, K^2156^ and E^2228^) and +1 (Tel1 L^2642^ and Y^2780^; Mec1 L^2254^ and Y^2361^). Inserts on the right show the electrostatic potential of surfaces around the −1 binding pocket for each kinase, *blue* indicating positive charge and red indicating negative charge. In all figures, the peptide substrate shown in yellow is superimposed from a structure of ATM bound to p53 (PDB ID 8OXO) with the original Leu at the −1 position is remodeled as Asp (*asterisk*). ATP loops of kinases are omitted to facilitate visualization. *Carbon* atoms are tan for Mec1 and *green* for Tel1. *D*, phosphoproteomic analysis showing that R2544A and N2616E mutations in the Tel1 substrate binding pocket selectively reduced phosphorylation of Tel1-dependent D/E-S/T phosphorylation sites, exerting no such effect on Tel1-dependent S/T-Q sites. Sites with ratios over 7 were capped at 7 for visualization purposes. *E*, yeast spot assay in which cells with the Tel1 double mutation show markedly increased sensitivity to DSB-inducing agent camptothecin (CPT) at a concentration of 30 micromolar. 4-fold dilution.

To investigate whether the D/E-S/T motif is a determinant for the phosphorylation of Tel1 substrates, we first chose to mutate this motif in a Rad50 site found to be Tel1-dependent, serine 469. The aspartic acid previous to serine 469 in Rad50 was mutated to an alanine, after which Rad50 itself was tagged with a 5xFLAG motif for immunoprecipitation (Fig. 6*A*). The phosphorylation of serine 469 was then quantified *via* immunoprecipitation mass spectrometry (IP-MS) using cells that featured wild-type Rad50 *versus* the motif-mutated Rad50. The amount of the unphosphorylated version of this peptide was found to be similar between both strains, but the amount of phosphorylated peptide was found to be drastically lower after motif mutation, strongly suggesting that the presence of acidic residues immediately preceding a serine or threonine is a strong determinant for Tel1-dependent phosphorylation (Fig. 6*B*).

To further validate the notion that Tel1 directly targets D/E-S/T sites, we turned to existing structural data comparing the substrate recognition pockets of Tel1 and Mec1. As shown in Figure 6*C*, the Mec1 pocket for recognition of substrate residues displays a negatively charged distribution near the −1 position, primarily due to the presence of an E^2228^ in its catalytic loop (Fig. 6*C*). In contrast, Tel1 features an asparagine (N^2616^) at this position, which, together with basic residues R^2544^ and H^2526^, creates a more positively charged distribution that is expected to favor acidic residues at the −1 position of substrates (Fig. 6*C*) (23, 24, 25). To test the prediction that Tel1 directly targets D/E-S/T motifs and that such targeting relies on the electrostatic determinants in the substrate recognition pocket, we mutated R^2544^ to alanine and N^2616^ to glutamic acid (hereafter name*d tel1-2mut)*, with the expectation that these mutations would specifically impact Tel1’s ability to target D/E-S/T motifs. We next performed a phosphoproteomic analysis comparing *tel1-2mut* to wild-type cells. Strikingly, phosphorylation of D/E-S/T and D/E-S/T-Q sites previously found to be Tel1-dependent were selectively attenuated in the double mutant, whereas Tel1-dependent S/T-Q sites lacking D/E at the −1 position showed no such attenuation (Fig. 6*D*). Additionally, the double mutant showed increased sensitivity to the topoisomerase I inhibitor camptothecin (CPT), suggesting an important role for R^2544^ and N^2616^ in the Tel1-mediated response to CPT-induced DNA damage (Fig. 6*E*). Ultimately, since the electrostatic manipulation of the substrate recognition pocket matched the predicted changes in substrate motif phosphorylation, these results strongly support the notion that D/E-S/T sites are directly phosphorylated by Tel1.

### A network of Tel1-dependent phosphorylation connects Tel1 to DNA damage responses and telomere maintenance

**Figure 7:**
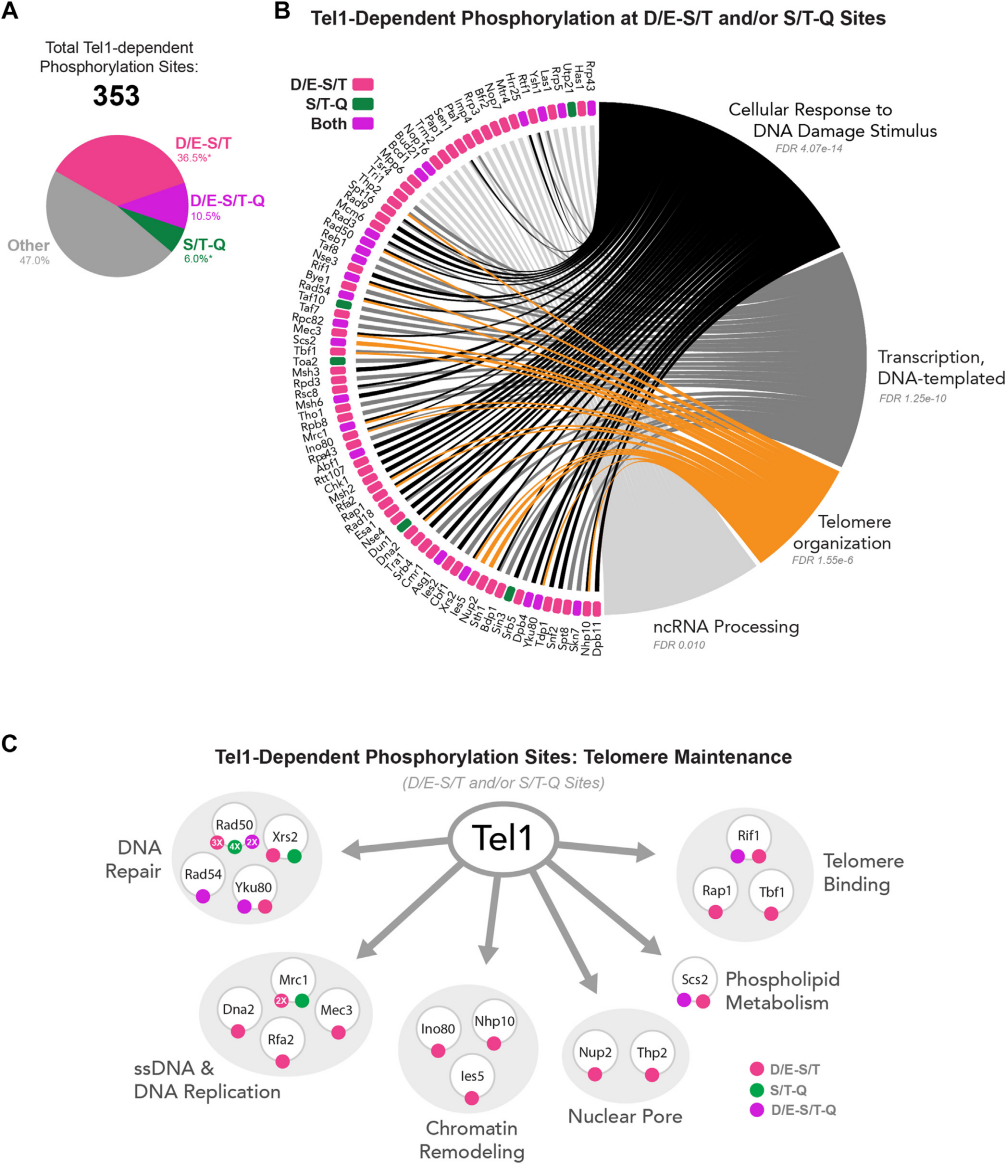
**An expanded network of Tel1-dependent phosphorylation targeting S/T-Q and D/E-S/T motifs.***A*, overview of Tel1-dependent phosphorylation events that were able to be localized and their motifs. ∗D/E-S/T and S/T-Q motif prevalence calculated excluding the combined motif, D/E-S/T-Q. *B*, Tel1-dependent phosphorylation events with either the S/T-Q or D/E-S/T motif implicated in the DNA damage response, DNA-templated transcription, telomere organization, or noncoding RNA processing. Proteins denoted as having “both” motifs either contain multiple sites with both motifs represented or at least one site with the combined motif, D/E-S/T-Q. *C*, breakdown of D/E-S/T and/or S/T-Q phosphorylation events implicated in telomere organization as denoted by *panelB*.

Overall, our deep coverage phosphoproteomic analysis identified 353 phosphorylation sites dependent on Tel1, of which about 53% are located at a D/E-ST, D/E-S/T-Q, or S/T-Q motif (Fig. 7*A*). Among proteins featuring these sites, some of the most enriched cellular processes are DNA damage response, transcription, and telomere organization. The majority of the proteins in these processes were phosphorylated at the D/E-S/T or D/E-S/T-Q motif, and have not previously been reported to be regulated by Tel1 phosphorylation (Fig. 7*B*). Given the established role of Tel1 in controlling telomere length, for which the functional Tel1 substrate(s) involved remain(s) unknown, we inspected Tel1-dependent phosphorylation in the set of proteins important for telomere maintenance (Fig. 7*C*). The result defines a cohesive network of proteins involved in DNA repair, including components of the MRX complex, as well as Rad54 and Yku80. Three telomere binding proteins intrinsically involved in telomere homeostasis also displayed Tel1-dependent phosphorylation, and represent likely effectors of Tel1 regulation at telomeres. Chromatin remodeling proteins linked to the Ino80 remodeller, which were previously reported to be involved in telomere maintenance, also had Tel1-dependent phosphorylation (26). Other categories included proteins involved in DNA replication, ssDNA transactions, and nuclear pores. Of importance, in the majority of the proteins involved in telomere maintenance found to have Tel1-dependent phosphorylation, the detected phosphorylation site resides at a D/E-S/T motif. These findings provide a framework of proteins and phosphorylation sites to guide functional and mechanistic analyses of Tel1 function and highlight the importance of this novel motif to expand our knowledge of the network of Tel1 signaling.

## Discussion

Tel1 has long been recognized as a key regulator of telomeres and DSB responses in budding yeast. The molecular mechanisms and specific substrates through which Tel1 controls these processes remain elusive. Here we mapped over 300 phosphorylation events dependent on Tel1, most of which are independent of the downstream kinases Rad53 and Dun1, and represent potential direct targets of Tel1. Consistent with these events being directly mediated by Tel1, we observed an enrichment in the canonical S/T-Q motif for PIKK phosphorylation. Surprisingly, we observed an even greater enrichment for a D/E-S/T motif, which we propose is a novel preferential motif for Tel1 phosphorylation. The identification of this motif and potential novel substrates and sites of Tel1 phosphorylation should provide a more comprehensive framework of the Tel1 signaling network for functional studies aiming at defining the molecular mechanism of Tel1 action.

Several lines of evidence are consistent with the D/E-S/T motif being directly targeted by Tel1. First, the D/E-S/T motif was the most enriched motif in the set of Tel1-dependent phosphorylation and was independent of known downstream kinases (and independent of any other kinase found to carry a Tel1-dependent phosphorylation). Second, we observed that the D/E-S/T-Q motif, which matches the canonical S/T-Q as well as the novel D/E-S/T motif, was the most enriched motif in the set of Tel1-dependent phosphorylation events, occurring at a frequency over 19-fold higher than in the set of Tel1-independent events. Third, analysis of the substrate binding pocket of Tel1 revealed that it contains a positively charged distribution that favors acidic residues at the −1 position of substrates. Fourth, the D/E-S/T preference is specific to Tel1 and absent from signaling mediated by Mec1, whose substrate binding pocket has a negatively charged distribution that does not favor acidic residues at the −1 position of substrates. Fifth, mutation of a highly Tel1-dependent D/E-S/T site on Rad50 to remove an acidic residue at the −1 position dramatically reduced the amount of phosphorylation observed after Tel1 activation with MMS. Sixth, mutation of the Tel1 substrate binding pocket that makes the electrostatics incompatible with an acidic residue at the −1 position of the substrate selectively reduced the phosphorylation of Tel1-dependent sites featuring the D/E-S/T motif. We believe that this last point is particularly compelling since manipulating the electrostatics of the pocket assumes direct interaction of the substrate binding pocket with the D/E-S/T motifs on the substrates. We note, however, that we cannot fully eliminate the possibility that the identified D/E-S/T motif is being caused indirectly by another kinase downstream of Tel1. Since we were unable to obtain active recombinant Tel1 preparations that were devoid of contaminating kinase activity, we could not confirm the direct phosphorylation of the D/E-S/T motif using *in vitro* kinase reactions.

The finding of a non-canonical mode of Tel1 signaling will help us understand how Tel1 plays specific roles in telomere maintenance and DNA repair that are not shared with Mec1. Since Tel1 and Mec1 have been reported to share similar substrate specificity toward the S/T-Q motif, and redundantly target many of the same proteins (12, 13), it has been difficult to determine the basis of Tel1’s unique biological functions. We propose that the specificity of Tel1 signaling in targeting D/E-S/T motifs helps explain the specialized roles of Tel1 in the control of telomere length and other biological processes.

The ability of PIKKs to target non-S/T-Q motifs has been recently documented in human cells. For example, we recently found that DNA-PKcs can directly phosphorylate an S/T-bulky-D/E motif (27). Additionally, ATR was previously found to auto-phosphorylate at an S/T-P motif (28). While we have not observed an enrichment of the D/E-S/T motif in a set of ATM-dependent events we recently reported, we do not exclude the possibility that ATM may also target such a motif, or other non-S/T-Q motifs (27). More in-depth analysis of ATM-dependent signaling in mammalian cells will be needed to properly probe alternative motifs.

## Experimental procedures

### Yeast strains and culture

A complete list of yeast strains used in this study can be found in Table S2. ORF deletions were performed *via* the established polymerase chain reaction (PCR)-based strategy to amplify resistance cassettes with flanking sequence homologous to a target gene (29). All ORF deletions were verified by PCR with one set of primers targeting the wild-type sequence and one set of primers targeting the null DNA sequence. Yeast strains were grown at 30 °C in a shaker incubator set to 220 rpm. For strains with integrated genetic modifications, YEP-D media was used. For SILAC cultures, yeast strains were grown in -Arg -Lys media supplemented with either isotopically normal arginine and lysine or the ^13^C^15^N isotopologues. Excess proline to prevent arginine-to-proline conversion was added to SILAC media at a concentration of 80 mg/L.

### CRISPR mutation

The Hrr25as, *tel1-2mut*, and Rad50 D468A strains were created by first ligating duplexed primers containing the sequence for gRNA targeting sequences corresponding to Hrr25 I82, Tel1 R2544 and N2616, and Rad50 D468 into CRISPR-Cas9 vectors. Candidate gRNA sequences were identified using CHOPCHOP (30, 31, 32). These plasmids were transformed into yeast along with gBlock DNA segments containing the corresponding mutations, after which transformants were plated on -Leu to select for yeast expressing the plasmid. After 2 days, colonies were selected and submitted for Sanger sequencing to determine whether the mutation occurred. Positive clones were then cultured and plated sparsely on YPD agar before being stamped on -Leu to select for clones that had lost the CRISPR-Cas9 plasmid.

### Spot assays

For dilution spot assays, 5 ml of yeast culture were grown to saturation at 30 °C. One OD600 equivalent of the saturated culture was then 4- or 5-fold (specified in figure legends) serially diluted in a 96-well plate in sterile water. Dilutions were then spotted onto agar plates with or without treatments as described in the figure legends using a bolt pinner.

### Phosphoproteomics

For large-scale phosphoproteomics experiments, 300 ml SILAC cultures were grown in heavy or light SILAC media to mid-log phase and treated as described in the figure legends. Normal-scale experiments utilized 150 ml SILAC cultures. Cells were pelleted at 1000 rcf and washed with Tris-EDTA buffer containing 1 mM PMSF. Pellets were lysed by bead beating with 0.5 mm glass beads for three cycles of 10 min with 1 min rest time between cycles at 4 °C in lysis buffer (150 mM NaCl, 50 mM Tris pH 8.0, 5 mM EDTA, 0.2% Tergitol type NP-40) supplemented with complete EDTA-free protease inhibitor cocktail (Pierce), 5 mM sodium fluoride, and 10 mM *β*-glycerophosphate. 12 mg of each light- and heavy-labeled protein lysate was pooled, and the pooled lysates were then denatured and reduced with 1% SDS and 5 mM DTT at 42 °C, and then alkylated with 25 mM iodoacetamide. Lysates were mixed with cold PPT solution (49.9% EtOH, 50% acetone, 0.1% acetic acid) to precipitate on ice for 30 min, after which the precipitated protein was pelleted *via* centrifugation at 4000 rcf. Pellets were washed once with PPT and then resuspended in Urea/Tris solution (8M urea, 50 mM Tris pH 8.0). The urea-solubilized pellet was then diluted to 1M urea using NaCl/Tris solution (150 mM NaCl, 50 mM Tris pH 8.0) and digested overnight at 37 °C with TPCK-treated trypsin. Phosphoenrichment for each replicate was performed using two Thermo Fisher High Select Fe-NTA phosphopeptide enrichment kits (cat# A32992) according to the manufacturer’s protocol. Purified phosphopeptides were then extensively fractionated using HILIC chromatography on an Ultimate 3000 HPLC (40 fractions collected for large-scale, 20 for normal-scale), after which fractions were dried *via* vacuum concentration, resuspended in 0.1% TFA, and subjected to LC-MS/MS analysis on a Thermo Fisher Q-Exactive HF mass spectrometer using 70 min reverse-phase C18 gradients.

### IP-MS

For IP-MS experiments, yeast strains were grown to the mid-log phase and treated as described in the figure legends. Cells were pelleted at 1000G and washed with TE buffer containing 1 mM PMSF. Pellets were lysed as described for phosphoproteomics, after which lysates were incubated with antibody-conjugated agarose resin for 1 h at 4 °C. The resin was washed 4 times with lysis buffer. Elution was performed with IP elution buffer (1% SDS, 100 mM Tris pH 8.0). Elutions were brought to 10 mM DTT and alkylated with 25 mM iodoacetamide for 15 min at room temperature. Protein was precipitated and digested with trypsin as described for phosphoproteomics, after which C18 desalting was performed with a 50 mg Waters Sep-Pak column. Desalted peptides were resuspended in 0.1% TFA and subjected to LC-MS/MS analysis on a Thermo Fisher Q-Exactive HF mass spectrometer using 70 min reverse-phase C18 gradients for data-dependent acquisition followed by parallel reaction monitoring (PRM) analysis with the same gradient. MS methods files containing all instrument parameters utilized are included as supporting information.

### Mass spectrometry data analysis

Analysis of phosphoproteomic data was performed by first searching MS/MS spectra using the Comet search engine (part of the Trans Proteomic Pipeline; Seattle Proteome Center) over a composite yeast protein database consisting of both the normal yeast protein sequences downloaded from the *Saccharomyces* Genome Database (SGD) and their reversed protein sequences to serve as decoys and estimate the false discovery rate (FDR) in search results (33). Search parameters allowed for semi-tryptic peptide ends, a mass accuracy of 10 ppm for precursor ions, variable modifications for SILAC lysine and arginine (8.0142 and 10.00827 Da, respectively), variable modification for STY phosphorylation (79.966331 Da), and a static mass modification of 57.021465 Da for alkylated cysteine residues. Phosphorylation site localization probabilities were determined using PTMProphet, with SILAC quantification of identified phosphopeptides being performed using XPRESS with manual adjustment of mass tolerance in cases of isobaric interference (both modules are part of the Trans Proteomic Pipeline; Seattle Proteome Center) (34, 35). Tel1 SILAC phosphoproteomic data was subject to Bowtie filtering as previously described in Faca *et al.* 2020 (17). Processed phosphoproteomic data is available in Table S2.

Analysis of targeted parallel reaction monitoring (PRM) data was performed by first curating a spectral library in Skyline (MacCoss Lab) using data from both phosphoproteomic experiments and data-dependent acquisition analyses of IP samples (36). PRM data files (converted to mzXML format using Proteowizard msConvert) were then imported into Skyline featuring a target list that included phosphopeptides of interest on RAD50. A Skyline document containing all peptide and transition settings used is uploaded as part of the supporting information.

### Protein structure analysis

Structures for Tel1 (PDB ID 6S8F) and Mec1 (PDB ID 6Z2X) were aligned in PyMol while superimposing a p53 peptide substrate from a structure of human ATM bound to this peptide (PDB ID 8OXO).

## Data availability

All mass spectrometric data generated for this study are available through PRIDE *via* PXD identifier PXD053305 (https://www.ebi.ac.uk/pride/).

## Supporting information

This article contains supporting information.

## Conflict of interest

The authors declare that they have no conflicts of interest with the contents of this article.

## Figures and Tables

**Figure S4:**