# Supplementary material for: Profiling Tel1 signaling reveals a non-canonical motif targeting DNA repair and telomere control machineries
Source: J Biol Chem. 2025 Jan 16;301(3):108194. doi: 10.1016/j.jbc.2025.108194 (PMC11875207; doi:10.1016/j.jbc.2025.108194)
Supplement: Supplemental figure legend [file mmc1.docx]

**Supporting Figures Legend**

**Figure S1:** **Tel1-dependent phosphorylation sites on components of the MRX complex.** Many sites featuring various motifs were found to be Tel1-dependent, with Rad50 featuring the most Tel1-dependent phosphorylation events of any protein in the dataset.

**Figure S2:** **Tel1-dependent S/T-Q phosphorylation events featured on proteins involved in transcription.** A subnetwork of proteins annotated with Uniprot keywork “Transcription” was found via STRING network analysis among Tel1-dependent S/T-Q phosphorylation events.

**Figure S3:** **Tel1-dependent D/E-S/T phosphorylation event “RNA Metabolic Process” subnetwork.** Many of these phosphorylation events were found to be on proteins involved in RNA processing in addition to the DNA damage response, with a prominent cluster of proteins involved with ribosomal RNA processing specifically.

**Figure S4:** **Hrr25 Analog-sensitive mutant (Hrr25as1) shows sensitivity to ATP analog 1NM-PP1 and increased sensitivity to genotoxin MMS.** (A) Upon addition of 1.5uM 1NM-PP1 to YPD agar, an analog-sensitive mutant of essential kinase Hrr25 shows attenuated growth. (B) Hrr25as1 becomes sensitive to 0.006% MMS upon addition of a low dose of 1NM-PP1 (1.0uM). 5-fold dilutions.
